# The Intricate Connection between Bacterial α-Diversity and Fungal Engraftment in the Human Gut of Healthy and Impaired Individuals as Studied Using the In Vitro SHIME^®^ Model

**DOI:** 10.3390/jof9090877

**Published:** 2023-08-26

**Authors:** Benoît Marsaux, Frédéric Moens, Massimo Marzorati, Tom Van de Wiele

**Affiliations:** 1 ProDigest B.V., Technologiepark-Zwijnaarde 82, 9052 Ghent, Belgium; frederic.moens@prodigest.eu (F.M.); massimo.marzorati@prodigest.eu (M.M.); tom.vandewiele@ugent.be (T.V.d.W.); 2 Center for Microbial Ecology and Technology (CMET), Ghent University, Coupure Links 653, 9000 Ghent, Belgium

**Keywords:** SHIME^®^, human gastrointestinal tract in vitro model, mycobiome, microbiome, fungi–bacteria interactions, α-diversity

## Abstract

From the estimated 2.2 to 3.8 million fungal species existing on Earth, only a minor fraction actively colonizes the human gastrointestinal tract. In fact, these fungi only represent 0.1% of the gastrointestinal biosphere. Despite their low abundance, fungi play dual roles in human health—both beneficial and detrimental. Fungal infections are often associated with bacterial dysbiosis following antibiotic use, yet our understanding of gut fungi–bacteria interactions remains limited. Here, we used the SHIME^®^ gut model to explore the colonization of human fecal-derived fungi across gastrointestinal compartments. We accounted for the high inter-individual microbial diversity by using fecal samples from healthy adults, healthy babies, and Crohn’s disease patients. Using quantitative Polymerase Chain Reaction and targeted next-generation sequencing, we demonstrated that SHIME^®^-colonized mycobiomes change upon loss of transient colonizers. In addition, SHIME^®^ reactors from Crohn’s disease patients contained comparable bacterial levels as healthy adults but higher fungal concentrations, indicating unpredictable correlations between fungal levels and total bacterial counts. Our findings rather link higher bacterial α-diversity to limited fungal growth, tied to colonization resistance. Hence, while healthy individuals had fewer fungi engrafting the colonic reactors, low α-diversity in impaired (Crohn’s disease patients) or immature (babies) microbiota was associated with greater fungal abundance. To validate, antibiotic-treated healthy colonic microbiomes demonstrated increased fungal colonization susceptibility, and bacterial taxa that were negatively correlated with fungal expansion were identified. In summary, fungal colonization varied individually and transiently, and bacterial resistance to fungal overgrowth was more related with specific bacterial genera than total bacterial load. This study sheds light on fungal–bacterial dynamics in the human gut.

## 1. Introduction

It is estimated that Earth harbors between 2.2 to 3.8 million fungal species, spanning diverse ecological niches [1]. It is, therefore, not surprising that fungi have colonized every site of the human body. Within the human colonic microbiota, fungi coexist alongside bacteria, archaea, protozoa, and viruses. The human gastrointestinal tract (GIT) houses the highest microbial density, encompassing thousands of microbial strains [2]. While bacteria account for the majority of biomass [3,4], fungi constitute only 0.1% of the GIT biosphere, despite their global prevalence [5]. Indeed, many environmental fungi are not adapted to thrive in the human GIT due to its unique stressors, leading to a tripartite selection process. Environmental pressures like pH, oxygen levels, temperature, nutrient availability, bowel flow rates, water activity, and host secretions primarily shape this selection [6]. It is secondarily impacted by the ecological environment, whereby microbe–microbe interactions range from mutualism to competition [7]. The third layer of selection is imposed by the host’s innate and adaptive immune system, tasked with monitoring and mitigating risks to host health [8].

Each individual possesses a distinct microbial signature resulting from their own selective pressures. This diversity contributes to significant high inter-individual variability. Furthermore, the diurnal fluctuations in environmental and ecological conditions within the human GIT foster temporal shifts in the composition and function of the colonic microbiota [9]. The considerable inter- and intra-individual variability has precluded the identification of a core colonic fungal microbiota. Nonetheless, Ascomycota and Basidiomycota emerge as the prevailing fungal phyla across populations [10,11]. At genus level, prevalent fungi in human feces include *Candida*, *Saccharomyces*, *Galactomyces*, *Penicillium*, *Aspergillus*, *Malassezia*, and *Debaryomyces* [12,13].

Distinct fungi exhibit varied behaviors in the GIT. Some are transient, whereby they are present in human feces but do not actively colonize the GIT and, hence, only pass through [14]. Their presence in the colon is the result of frequent exposure from the environment (e.g., air or fermented food). Others are GIT residents, having adapted to its specific physiological conditions. These residents can be categorized as commensals, symbiotic with their host and integrated into the ecological network, or as opportunists requiring specific circumstances to thrive.

In healthy individuals, the resident colonic microbes prevent their growth through a phenomenon termed “colonization resistance” [15]. This colonization resistance intrinsically involves microbe–microbe interactions, such as competition for nutrients, niches and binding sites, and the release of antimicrobial agents [15]. Alterations in these interactions can lead to the emergence of opportunistic microbes. As compared to the commensals, opportunists rely on favorable environmental and ecological conditions which are often found in vulnerable patients [16]. The dichotomy between eubiosis, where opportunists face maximal colonization resistance, and dysbiosis, an altered state favoring opportunists, represents a crucial determinant of gut health [15]. Therefore, dysbiosis is associated with pathologies like inflammatory bowel diseases (IBD) and antibiotic-associated diarrhea [17,18].

Broad-spectrum antibiotics are a primary cause of dysbiosis, inducing critical shifts in colonic microbial composition, concentration, and function [19]. These changes provide opportunities for opportunistic microbes, including pathogens and pathobionts, to flourish. While the role of bacteria in these pathologies is increasingly understood [17,18], the impact of antibiotics on the human mycobiome remains largely uncharted territory, although antibiotic-treated mice have been found more susceptible to fungal infections [20].

Crucially, resident and transient microbes collectively contribute to host health, with the majority fostering synergistic interactions. Colonic bacteria play essential roles in enhancing gut integrity, modulating the intestinal epithelium [21], harvesting energy [22], protecting against pathogens [23], and regulating host immunity [24]. However, the fungal microbiota’s contribution to human health remains inadequately understood, partially due to uncertainty surrounding which fungal species actively colonize the human GIT. Techniques predominantly reliant on fecal samples hinder the differentiation between transient and resident microbes, creating a “black box” effect. To address this, controlled environments that exclude constant fungal introduction from the diet, such as in vitro models of the human GIT, are necessary. In addition, their use would enable mechanistic insights on fungi–bacteria interactions in healthy and impaired conditions, through sampling of the different colonic compartments in function of time.

The luminal and mucosal simulator of the human intestinal microbial ecosystem (L- and M-SHIME^®^, respectively) are complex, computer-controlled, in vitro models that have been instrumental in studying the colonic bacterial microbiome (features reviewed in [25]). They preserve microbial taxa representative of the lumen and mucus, with longitudinal differentiation between the proximal and distal colon, and maintain fecal donor metabolic phenotypes [25]. These models are particularly suited to look at the adaptation of the microbial ecosystem to changing environmental conditions, such as antibiotic treatments. Apart from probiotic yeast studies [26], the L- and M-SHIME^®^ have, thus far, not been used to study the fungal microbiome. Consequently, in this study we assessed the ability of the L- and M-SHIME^®^ to maintain the mycobiome abundance and diversity of the original donor, putting emphasis on discriminating between resident and transient fungi. Given the substantial inter-individual variability, we used fecal samples from both healthy adults and infants, and adult Crohn’s disease patients. By monitoring fungal and bacterial colonization kinetics across simulated human GIT conditions, both under eubiosis and antibiotic disturbance, we offer insights into fungal–bacterial interactions using a quantitative Polymerase Chain Reaction (qPCR) targeting the 18S rRNA gene and 16S rRNA gene, along with targeted ITS1 rRNA gene and 16S rRNA gene next-generation sequencing, respectively. This study pioneers the use of the L- and M-SHIME^®^ to explore bacterial–fungal interactions, setting the stage for a deeper understanding of fungi’s potential modulation of human health.

## 2. Materials and Methods

### 2.1. Chemical Products

All chemicals were obtained from Merck (Darmstadt, Germany) unless stated otherwise.

### 2.2. Sample Collection and Donor Descripion

Infant donors were selected based on the following inclusion criteria: healthy, age between 5 and6 months, no history of antibiotic nor pre- or probiotic intake, no constipation, and hospital borne. Samples were obtained from infant diapers. Healthy adults had no antibiotic nor pre- or probiotic dietary supplement intake in the previous 3 months, and no constipation. IBD patients were diagnosed with light-to-moderate Crohn’s disease, with flare-up symptoms. All samples were immediately transferred to a recipient containing an “Oxoid™ AnaeroGen™” bag to limit the samples’ exposure to oxygen and transferred to the lab for further use.

### 2.3. Experimental Design of Long-Term L and M-SHIME^®^

**Figure 1:**
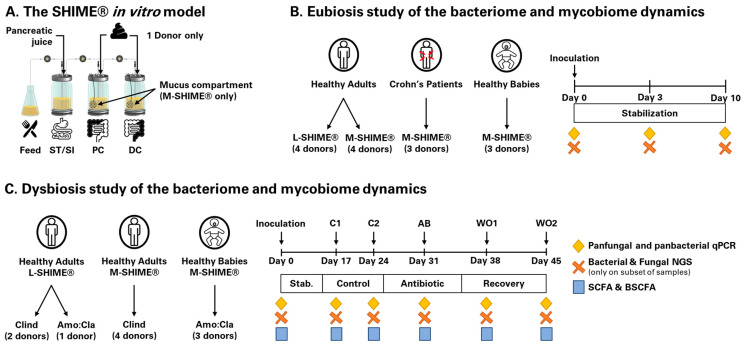
Schematic representation of the SHIME^®^ in vitro model (**A**); the experimental setup of the long-term eubiosis study (**B**) and dysbiosis study (**C**). ST/SI = stomach/small intestine; PC = proximal colon; DC = distal colon; M-SHIME^®^ = mucosal simulator of the human intestinal microbial ecosystem; L-SHIME^®^ = luminal simulator of the human intestinal microbial ecosystem; C1 and C2 = control period 1 and 2; AB = antibiotic period; WO1 and WO2 = wash-out period 1 and 2; Clind = clindamycin; Amo:Cla = amoxicillin:clavulanic acid mix; NGS = next-generation sequencing; SCFA = short-chained fatty acid; BSCFA = branched short-chained fatty acid; stab = stabilization.

Both L-SHIME^®^ and M-SHIME^®^ setups (ProDigest and Ghent University, Ghent, Belgium) were used in this study (Figure 1A). The baby M-SHIME^®^ [27] and the adult L-SHIME^®^ and M-SHIME^®^ [28] were performed as previously described, with feed composition adapted to mimic colonic nutrient composition of babies and adults. While the L-SHIME^®^ is an ideal technology to study luminal microbes, the M-SHIME^®^ allows a closer look at the interface between the mucosal and the luminal regions of the colon, and is, therefore, characterized by a different microbial community [29]. Importantly, as this study was exploratory in nature, there were no common donors between L-SHIME^®^ and M-SHIME^®^ reactors; we, therefore, did not assess the impact from having a mucosal compartment on microbial engraftment success from the same donor.

#### 2.3.1. Eubiosis Study

The eubiosis study consisted of monitoring the fungal and bacterial kinetics over 10 days without intervention using either an L-SHIME^®^ or an M-SHIME^®^. Briefly, upon inoculation of the SHIME^®^ reactors with fecal samples, the system receives three feeding cycles each day. During the stabilization period, the microbiota diversifies in each colonic compartment finally resulting in the presence of a stable microbial community. The latter contains the specific species that are resident in this colonic environment, whereas transient ones will be washed-out of the system. Hence, this period was used to discriminate between transient and resident fungal species. To account for inter-individual differences in microbial ecosystem composition, healthy babies (n = 3), healthy adults (n = 7), and Crohn’s disease patients in flare (n = 3) were investigated. The configuration of each SHIME^®^ is summarized in Figure 1B.

#### 2.3.2. Dysbiosis Study

The dysbiosis study consisted of monitoring the impact of antibiotic treatment on fungal and bacterial kinetics. The M-SHIME^®^ and L-SHIME^®^ experiments were performed as previously described [30]. Following an adaptation period of 14 days, the microbial diversity and concentration were monitored over a control period of two weeks (day 15 to day 28). This allowed us to determine the baseline microbial concentration, composition, and function. Subsequently, clindamycin (85 mg/L) (n = 2 adult L-SHIME^®^ and n = 4 adult M-SHIME^®^) or a 1:2 mix of amoxicillin:clavulanic acid (250 mg/L) (n = 1 adult L-SHIME^®^ and n = 3 baby M-SHIME^®^) was dosed in the proximal colon, three times per day, for seven days (day 29 to day 35). Considering the initial control period, any changes observed during this period can be attributed to the antibiotic treatment. This allows us to monitor whether resident fungal species can become opportunists upon the induction of dysbiosis of the colonic microbiota. Finally, the recovery of the microbial ecosystem from antibiotic disruption was followed for two weeks post-antibiotic cessation (day 36 to day 49). This was performed to further decipher the ability of opportunists to remain competitive against a recovering colonic microbiota. To account for different microbial populations, healthy babies and adults were investigated. The timeline is summarized in Figure 1C. Of notice, the microbial inocula obtained from the three healthy babies were also used in the eubiosis study.

### 2.4. Metabolic Analysis

To assess the impact of antibiotic treatment on the metabolic activity of the microbiome, short-chain fatty acid (SCFA, acetate, propionate, and butyrate) and branched short-chain fatty acid (BSCFA, isobutyrate, isovalerate, and isocaproate) were determined as previously described [31] on all samples of the dysbiosis study. Due to limited concentration of BSCFA, only SCFA data are reported in the manuscript.

### 2.5. Microbial Community Analysis by qPCR

Total bacterial and fungal concentrations were determined using a quantitative Polymerase Chain Reaction (qPCR). It was performed on samples collected at day 3 and day 10 during the eubiosis study, as well as samples collected at day 14, day 21, day 28, day 35, and day 42 during the dysbiosis study. In brief, DNA was isolated as described before [32] with minor modifications [33] from 1 mL luminal samples. Subsequently, qPCR was performed using a QuantStudio 5 Real-Time PCR system (Applied Biosystems, Foster City, CA, USA). Each sample was run in technical triplicate. For total bacterial concentration determination, the qPCRs were performed as described previously with the primers UNI-F (5′-GTGSTGCAYGGYYGTCGTCA-3′) and UNI-R (5′-ACGTCRTCCMCNCCTTCCTC-3′), which target the 16S rRNA gene [34]. Similarly, the fungal concentration determination was performed using qPCRs as described previously [35] with the primers FF390 (5′-CGATAACGAACGAGACCT-3′) and FR1 (5′-AICCATTCAATCGGTAIT-3′), which target the 18S rRNA gene, with, however, an elongation step at 72 °C for 30 s and a reaction mixture containing 0.5 µM of each primer. Results are reported as log_10_(16S rRNA gene copies/mL) and log_10_(18S rRNA gene copies/mL) for the total bacterial and fungal concentrations, respectively.

### 2.6. Microbial Community Analysis by 16S rRNA and ITS1 rRNA Gene-Targeted Sequencing

On a selection of donors, microbial community composition was assessed at all time points, from the proximal colon samples only. Samples were sent out to LGC Genomics GmbH (Berlin, Germany) for next-generation sequencing of both the 16S rRNA gene amplicons of the V3–V4 region for bacteria, and the ITS1 rRNA gene amplicons for fungi. For bacteria, the 341F (5′-CCTACGGGNGGCWGCAG-3′) and 785R (5′-GACTACHVGGGTATCTAAKCC-3′) primers were used [36]. For fungi, the ITS1F (5’-CTTGGTCATTTAGAGGAAGTAA-3’) [37] and ITS2 (5’-GCTGCGTTCTTCATCGATGC-3’) [38] primers were used.

In all cases, the amplification was performed using PCRs and included about 1–10 ng of DNA extract (total volume 1 µL), 15 pmol of each forward and reverse primer in 20 µL volume of 1× MyTaq buffer containing 1.5 units MyTaq DNA polymerase (Bioline GBmH, Luckenwalde, Germany), and 2 μL of BioStabII PCR Enhancer (Sigma-Aldrich Co., Saint-Louis, MO, USA). For each sample, the forward and reverse primers had the same 10 nt barcode sequence. PCRs were carried out for 35 cycles using the following parameters: 1 min 96 °C pre-denaturation, 96 °C denaturation for 15 s, 55 °C annealing for 30 s, 70 °C extension for 90 s, and hold at 8 °C.

The DNA concentration of amplicons of interest was assessed using gel electrophoresis. About 20 ng amplicon DNA of each sample were pooled for up to 48 samples carrying different barcodes. The amplicon pools were purified using one volume Agencourt AMPure XP beads (Beckman Coulter, Inc., Brea, CA, USA) to remove primer dimer and other small mispriming products, followed by an additional purification on MiniElute columns (QIAGEN GmbH, Hilden, Germany). About 100 ng of each purified amplicon pool DNA was used to construct Illumina libraries using the Ovation Rapid DR Multiplex System 1-96 (NuGEN Technologies, Inc., San Carlos, CA, USA). Illumina libraries (Illumina, Inc., San Diego, CA, USA) were pooled and size selected by preparative gel electrophoresis. Sequencing was performed on an Illumina MiSeq using V3 Chemistry.

### 2.7. Data Analysis

In case of total bacteria and total fungi concentrations, data were log-transformed because of their log-normal distribution. No statistical test was performed due to the small sample size.

Both 16S-rRNA-gene- and ITS1-rRNA-gene-targeted sequencing data were processed using the DADA2 R package according to the pipeline tutorial [39]. In a first quality control step, the primer sequences were removed and reads were truncated at a quality score cut-off (truncQ = 2). Besides trimming, additional filtering was performed to eliminate reads containing any ambiguous base calls or reads with high expected errors (maxEE = 2.2). After dereplication, unique reads were further denoised using the Divisive Amplicon Denoising Algorithm (DADA) error estimation algorithm and the selfConsist sample inference algorithm (with the option pooling = TRUE). The obtained error rates were inspected and after approval; the denoised reads were merged. Finally, the amplicon sequence variant (ASV) table obtained after chimera removal was used for taxonomy assignment using the Bayesian Classifier and either the DADA2 formatted Silva v138 [40] for bacteria or the UNITE 9 ([41], Version 17.10.2022) for fungi. The taxonomic assignment of sequences accounting for at least 1% of the total read count in at least one sample were confirmed using RDP classifier 2.12 with the 16S rRNA training set 18 [42] and the MycoBank pairwise alignment tool [43] for bacteria and fungi, respectively. Only sequences having at least 5 counts in one sample and assigned to bacteria kingdom for 16S rRNA gene or fungi kingdom for ITS1 rRNA gene sequencing were retained. A total of 1,442,780 and 2,302,166 withheld sequences were binned into amplicon sequence variants (ASVs).

Data at genus level were processed using the ProDigest Analytics Suite after applying a total sum normalization. Quantitative microbiome profiling was performed by correcting relative abundance with 16S rRNA and 18S rRNA gene qPCR data for bacteria and fungi, respectively. Diversity indices (observed species, Shannon’s, Simpson’s, and DAPC) were calculated using the ProDigest Analytics Suite. For the eubiosis study, a fungal:bacterial ratio was calculated for each donor group (healthy adults, healthy babies, and Crohn’s disease patients with flare-up symptoms) by dividing for each sample their 18S rRNA gene qPCR values by the average 16S rRNA gene qPCR values. Data were plotted using violin plots with median values and expressed as ‰(18S/16S rRNA gene copies/mL). For the dysbiosis study, volcano plots obtained through differential abundance analysis (treeclimbR), followed by pair-wise analysis was used to determine bacterial ASVs features for which the abundance was significantly different between samples with “low” versus “high” fungal concentrations. Principal component analysis (PCA) was performed using ClustVis (https://biit.cs.ut.ee/clustvis/ accessed on 13.04.2023) with parameters as previously described [44].

## 3. Results

### 3.1. Wash-Out of Microbes during Stabilization Period in Eubiosis Conditions

#### 3.1.1. Microbial Concentration in Eubiosis

Following inoculation of human fecal microbiota in SHIME^®^ setups, qPCR was used to monitor panbacterial and panfungal concentrations in proximal and distal colon compartments of SHIME^®^ setups during ten days of stabilization. Five healthy adults were used for separate inoculations of five L-SHIME^®^ experiments whereas three healthy adults, three healthy babies, and three diagnosed Crohn’s disease patients with flare-up symptoms were used to separately inoculate nine M-SHIME^®^ experiments. To account for experimental reproducibility, healthy adults 5 (n = 5), 6 (n = 2), 7 (n = 3), and 8 (n = 8) were used multiple times in parallel experiments.

**Figure 2:**
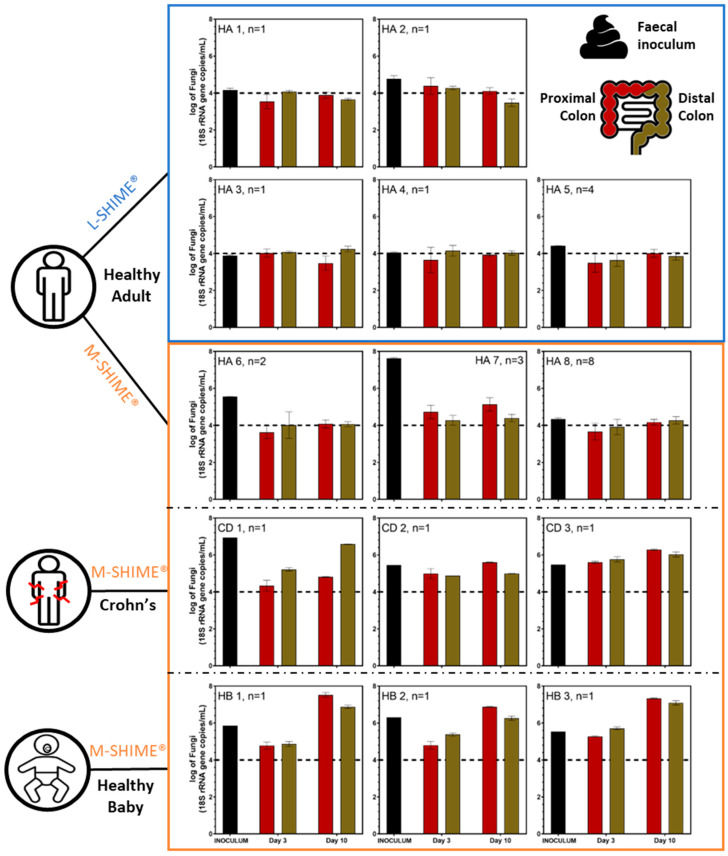
Total fungal concentration in the fecal inocula and during stabilization (day 3 and day 10) in the SHIME^®^ in eubiosis conditions, measured using qPCR targeting the 18S rRNA gene region and expressed in log_10_(18S rRNA gene copy/mL). The number of biological replicates for each donor is indicated by the n values, and the analysis was performed using technical triplicates of measurement. The limit of quantification is indicated by a black dashed line. HA = healthy adults; CD = Crohn’s disease patient with flare-up symptoms; HB = healthy babies.

**Figure 3:**
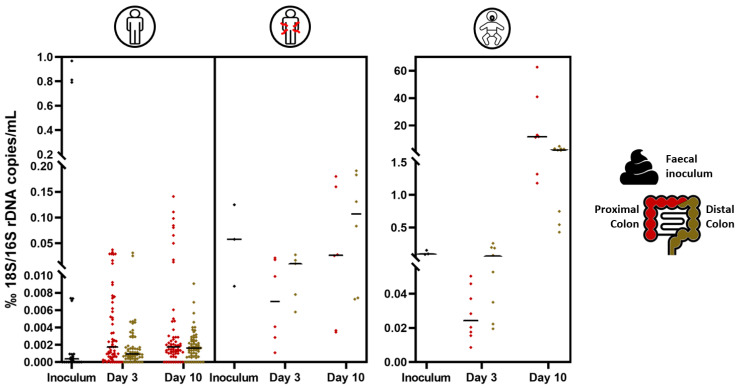
Violin plot of the fungal:bacterial concentration ratio in healthy adults, Crohn’s disease patients with flare-up symptoms, and healthy babies in the fecal inocula and during stabilization (day 3 and day 10) in the SHIME^®^ in eubiosis conditions, expressed in ‰ 18S/16S rRNA gene copy/mL. Median values are indicated with a black straight line.

The fungal concentration in the fecal inocula of healthy adults strongly varied between individuals, ranging from 3.88 to 7.62 log_10_(18S rRNA gene copy/mL) (Figure 2). Interestingly, most Crohn’s patients [ranging from 5.45 to 6.94 log_10_(18S rRNA gene copy/mL)] and healthy babies [ranging from 5.53 to 6.31 log_10_(18S rRNA gene copy/mL)] displayed higher fecal fungal levels than healthy adults [ranging from 3.88 to 5.55 log_10_(18S rRNA gene copy/mL)], with the exception of healthy adult 7 [i.e., 7.62 log_10_(18S rRNA gene copy/mL)] who harbored the highest fungal load. Interestingly, the bacterial concentration was highly consistent across the fecal inocula of all healthy adults [mean 10.69 ± 0.13 log_10_(16S rRNA gene copy/mL)] (Figure S1). Crohn’s patients 1 and 2 displayed similar bacterial levels to healthy adults. In contrast, Crohn’s disease patient 3 and the three healthy babies had a one-log bacterial concentration reduction [mean 9.83 ± 0.23 log_10_(16S rRNA gene copy/mL)]. Overall, qPCR analysis revealed the fungal:bacterial ratio to be about 25- to 50-fold higher in Crohn’s disease patients and healthy babies as compared to healthy adults, respectively (Figure 3).

Following inoculation of the SHIME^®^ experiments, fungi reached similar levels in the in vitro model for both the proximal and distal colon reactors regardless of the donor or the presence of a mucosal compartment in the SHIME^®^, except for Crohn’s disease patient 1 (Figure 2). Throughout the stabilization period, the fungal level decreased for all healthy adults, especially healthy adult 7, reaching concentrations close to the limit of quantification. In sharp contrast, fungal levels in healthy babies and Crohn’s disease patients during the stabilization period remained similar to their respective fecal inoculum (i.e., Crohn’s disease patients 1, 2, and 3, and healthy baby 2) or even increased by day 10 (i.e., healthy babies 1 and 3). In terms of bacterial concentrations, an overall decrease in bacterial levels (compared to the fecal inocula) was observed after ten days in the SHIME^®^. A similar level of bacteria was found across all adult donors. Interestingly, the adults’ bacterial concentrations in both colonic regions were higher than those of the three healthy babies’ ones regardless of health status and the presence of a mucosal compartment (Figure S1). During all time points of the stabilization period, the SHIME^®^ displayed consistent fungal:bacterial ratios in both colonic reactors, similar to those observed in the respective fecal inocula for healthy adults and Crohn’s disease patients. Similar results were obtained for healthy babies at day 3; yet, the fungal:bacteria ratio largely increased by day 10 (Figure 3). These observations were experimentally reproducible across multiple SHIME^®^ experiments where the same fecal inoculum was used, as exemplified with healthy adults 5, 6, 7, and 8 that were used to inoculate different SHIME^®^ experiments (Figure 2 and Figure S1).

#### 3.1.2. Quantitative Microbiome Profiling and Microbial Diversity Dynamics in Eubiosis

Illumina next-generation sequencing targeting the ITS1 rRNA gene and the 16S rRNA gene regions was performed to analyze the mycobiome and the bacteriome profiles on a selection of donors, respectively. Sequence counts were corrected using qPCR data to account for the different microbial loads in between tested subjects. Healthy adults 1 and 7 were selected to discriminate between healthy adults containing a low and high fungal concentration in the fecal inoculum, respectively. Crohn’s disease patient 2 and healthy baby 3 were selected as representatives of their respective donor groups. Only the proximal colon samples were sequenced as similar fungal concentration was reached between the proximal and distal colon reactors.

**Figure 4:**
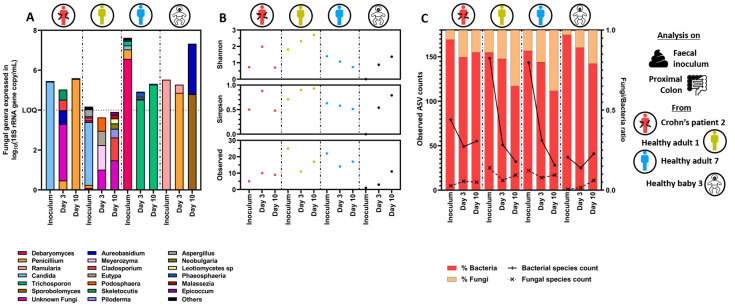
Fungal quantitative microbiome profiling (**A**), α-diversity indexes (**B**), and fungal:bacterial ASV ratio (**C**) in fecal samples and resulting proximal colon reactors during stabilization in eubiosis conditions. For the fungal quantitative microbiome profiling, each bar represents the total fungal concentration in the sample, whereas the colors represent the proportion of each fungal genera. ASV = amplicon sequence variant.

During the stabilization period, the mycobiome profiles drastically shifted in the proximal colon reactors as compared to the initial composition present in the fecal samples. This was observed for all donors and was characterized by a fewer number of amplicon sequence variants (ASVs) (Figure 4A,B). Briefly, the fecal inoculum of healthy adult 1 was dominated by *Candida* (ASV closely related to *C. albicans*) whereas a more diverse but changing community composition was observed at day 3 and day 10 of the stabilization period. While the fecal inoculum of healthy adult 7 was dominated by *Debaryomyces* (ASV closely related to *D. hansenii*), the proximal colon mycobiome became dominated by *Trichosporon* at day 3 and day 10 of the stabilization period. The fecal inoculum of Crohn’s disease patient 2 was dominated by *Candida* (ASV closely related to *C. albicans*), which shifted towards a more diverse fungal community at day 3 and became dominated by *Penicillium* at day 10 of stabilization. Finally, the fecal inoculum of healthy baby 3 was dominated by *Ramularia*, whereas the mycobiome in the proximal colon reactors became intermediately dominated by *Penicillium* at day 3 but dominated by *Aureobasidium* and *Sporobolomyces* at day 10 of the stabilization period. As a result of these mycobiome shifts, the α-diversity was reduced with time in healthy adult 7, while it remained unchanged in Crohn’s disease patient 2. By contrast, the mycobiome α-diversity of healthy adult 1 and healthy baby 3 became enriched and more evenly distributed with time.

In contrast, the fecal bacteriome was more diverse than the mycobiome, with 10-fold higher bacterial ASVs than fungal ASVs on average, and the profiles remained stable between day 3 and day 10 of the SHIME^®^ experiments (Figure S2A–C and Figure 4C). This is confirmed by every α-diversity indicator remaining largely unchanged between day 3 and day 10 across all donors, and samples clustering per donor. However, the number of observed ASVs present in the fecal inoculum strongly decreased in the proximal colonic reactors after three days of stabilization. This was especially the case in both healthy adults which were characterized by a 4-fold loss in observed ASVs.

### 3.2. Fungal Overgrowth Following Bacterial Diversity Reduction during Dysbiosis Induced with Different Antibiotic Treatments

Fungal pathologies often coincide with antibiotic use. We, therefore, investigated the impact of two antibiotics (i.e., mix of amoxicillin:clavulanic acid or clindamycin) on a stable microbiome community using the L-SHIME^®^ or the M-SHIME^®^. Different healthy donors (babies and adults) were studied to account for inter-individual differences. Only the experiment using the three healthy babies is a direct follow-up of the stabilization study (Section 3.1). To account for experimental reproducibility, healthy adults 9 (n = 3), 10 (n = 3), and 11 (n = 2) were used multiple times in parallel experiments.

#### 3.2.1. Microbial Concentration during Eubiosis and the Antibiotic Treatment Period

Bacterial and fungal concentrations were evaluated using qPCR at different time points: two during the control period (=baseline values), one during the antibiotic treatment period, and two during the wash-out period (=recovery).

**Figure 5:**
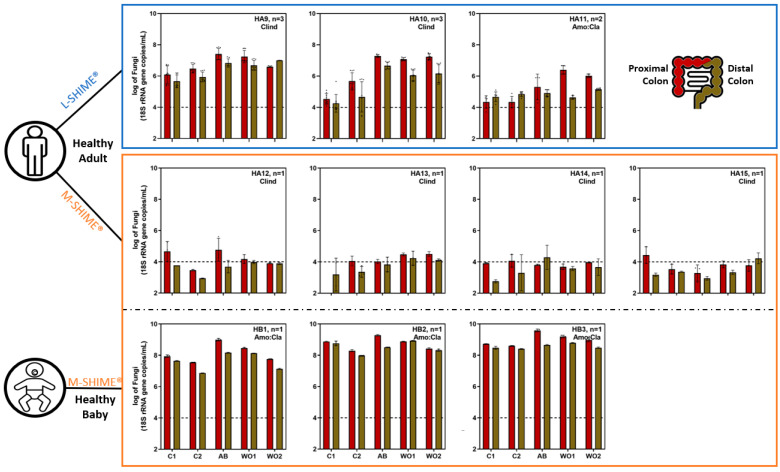
Total fungal concentration in the SHIME^®^ during eubiosis conditions (control period = C1, C2), during antibiotic treatment (=AB) and during the recovery period (wash-out = WO1, WO2) measured using qPCR targeting the 18S rRNA gene region and expressed in log_10_(18S rRNA gene copy/mL). The number of biological replicates for each donor is indicated by the n values, and the analysis was performed using technical triplicates of measurement. The limit of quantification is indicated by a black dashed line. HA = healthy adults; HB = healthy babies; Clind = clindamycin; Amo:Cla = amoxicillin:clavulanic acid mix.

Firstly, during the control period the fungal and bacterial concentration was either similar or higher in the proximal than the distal colon, independent of the presence of a mucosal compartment and the donor studied (Figure 5 and Figure S3). The colon reactors of the SHIME^®^ experiments inoculated with healthy adults’ fecal samples were characterized by low fungal concentrations with values close to the limit of quantification. The only exception was for the colonic reactors inoculated with the fecal microbiota from healthy adult 9 that had about a 2.3-log_10_ higher fungal load. In contrast, the colonic reactors inoculated with the fecal samples of the three healthy babies had high fungal concentration (mean 8.19 ± 0.56 log_10_(18S rRNA gene copy/mL)). Second, the bacterial concentrations for healthy adults were higher in the reactors of the L-SHIME^®^ experiments (mean 9.74 ± 0.32 log_10_(16S rRNA gene copy/mL)) as compared to the M-SHIME^®^ experiments (mean 9.00 ± 0.23 log_10_(16S rRNA gene copy/mL)). The bacterial concentration in the reactors of the experiments inoculated with the fecal samples of the three healthy babies fell in the same range as those inoculated with the healthy adults (mean 9.51 ± 0.23 log_10_(16S rRNA gene copy/mL)).

Upon antibiotic treatment, fungal concentrations strongly increased during the experiments that had evident fungal colonization during the control period, regardless of the antibiotic tested (Figure 5). For the experiments inoculated with the microbiota from healthy adults, the fungal overgrowth happened either during the antibiotic treatment or the wash-out period, remaining stable afterwards. By the end of the wash-out period, healthy adults 9, 10, and 11 had a 0.9-log_10_, 1.6-log_10_, and 1.9-log_10_ increase in the proximal colon reactors as compared to the control period, respectively. Healthy babies 1, 2, and 3 also displayed a 1.4-log_10_, 1.0-log_10_, and 1.0-log_10_ higher fungal level during antibiotic treatment in the proximal colon reactors, respectively. Upon cessation of the antibiotic treatment, the values returned to their initial level by the end of the wash-out period. In contrast, during the experiments performed with healthy adults 12, 13, 14, and 15, that were all characterized by values below the limit of quantification during the control period, no increase in fungal concentration was observed upon antibiotic treatment. In addition, bacterial concentration remained largely unchanged upon antibiotic treatment in most healthy adults, except for healthy adult 13. However, bacterial concentrations strongly decreased during the experiments performed with the three healthy babies (Figure S3). Upon wash-out of the antibiotic, the bacterial concentration recovered towards its initial level.

#### 3.2.2. Short-Chain Fatty Acid Production during Eubiosis and the Antibiotic Treatment Period

Acetate, propionate, and butyrate concentrations were quantified at all time points for all tested donors. In addition, branched short-chain fatty acids (i.e., isocaproate, isobutyrate, and isovalerate), caproate, and valerate concentrations were quantified, with values continuously low throughout the experiment. Because short-chain fatty acid (SCFA) production was similar across the simulated proximal and distal colons, and the impact of antibiotic treatment was stronger in the simulated proximal colon, only the results of the latter will be discussed in this section, while the simulated distal colon results can be consulted in supplementary (Figure S4).

**Figure 6:**
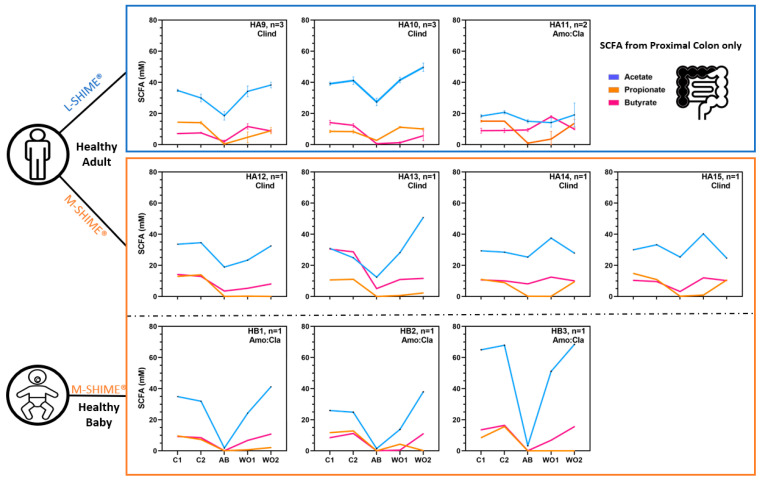
Acetate, propionate, and butyrate concentrations in the proximal colon reactors during eubiosis conditions (control period = C1, C2), during antibiotic treatment (=AB), and during the recovery period (wash-out = WO1, WO2). The number of biological replicates for each donor is indicated by the n values, and the analysis was performed with single measurement. HA = healthy adults; HB = healthy babies; Clind = clindamycin; Amo:Cla = amoxicillin:clavulanic acid mix; SCFA = short-chain fatty acid.

The concentration and the ratio between the three major SCFA were donor-dependent, and their values remained largely stable during the control period (Figure 6). Both tested antibiotic treatments strongly impacted SCFA concentrations, thereby reducing inter-individual differences. Indeed, acetate (c.a., 20 mM), propionate (c.a., <5 mM), and butyrate (c.a., <10 mM) levels were similar between all experiments performed with healthy adults, while only limited acetate (c.a., <5 mM) was produced during those inoculated with healthy babies. Importantly, the antibiotic treatments especially impacted propionate production in the proximal colonic reactors inoculated with healthy adults as propionate accounted for, c.a., 23% total SCFA prior antibiotic treatment as opposed to, c.a., 0.01% total SCFA during antibiotic treatment, when grouping all reactors together per time points. In contrast, butyrate accounted for, c.a., 22% total SCFA prior antibiotic treatments and, c.a., 19% total SCFA during antibiotic treatment. Upon ceasing antibiotic dosage, SCFA concentration increased again, especially acetate, whereas butyrate and propionate recovery was donor dependent. In particular, the SCFA profiles shifted from higher butyrate production to higher propionate production for healthy adult 10, whereas the opposite was observed for healthy adult 9 and 15. The butyrate production was recovered for all healthy babies, but the propionate production was not.

#### 3.2.3. Quantitative Microbiome Profiling and Microbial Diversity Dynamics during Eubiosis and the Antibiotic Treatment Period

The mycobiome and bacteriome profiles, corrected using qPCR data to account for microbial load differences, were studied for a selection of donors. The experiments performed with the fecal inocula of healthy adults 10 and 11 were selected because they were characterized by an increased fungal concentration during the antibiotic treatment. The experiment performed with healthy adult 12 was selected due to the consistent low fungal concentrations present in the reactors. The healthy baby 3 experiment was selected as a representative of its group. The simulated proximal colon presented the highest fungal concentration and was, therefore, analyzed.

**Figure 7:**
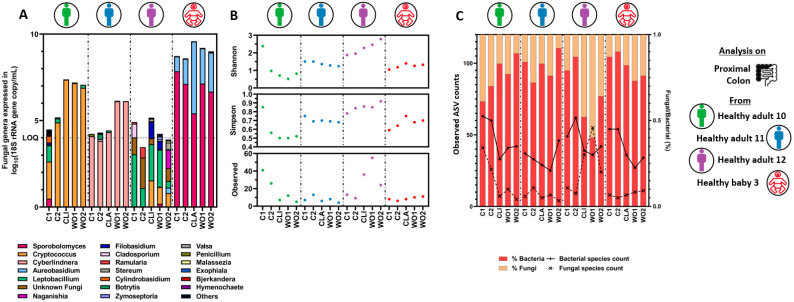
Fungal quantitative microbiome profiling (**A**), α-diversity indexes (**B**), and fungal:bacterial ASV ratio (**C**) in the proximal colon reactors during eubiosis conditions (control period = C1, C2), during antibiotic treatment (clindamycin (=CLI) or a mix of amoxicillin:clavulanic acid (=CLA), and during the recovery period (wash-out = WO1, WO2). For the fungal quantitative microbiome profiling, each bar represents the total fungal concentration in the sample, whereas the colors represent the proportion of each fungal genera. ASV = amplicon sequence variant.

First, only few fungi dominated the mycobiome of each donor during the control period (Figure 7A). These fungi remained dominant throughout the experiment showing that the antibiotic treatment only contributed to their expansion but had no impact on the fungal diversity. This is confirmed by most α-diversity indicators remaining constant, except for healthy adult 12 that was characterized by a fungal concentration close to the limit of quantification and likely more impacted by sequence artefacts (Figure 7A,B). Specifically, the experiment performed with healthy adult 10 was dominated by *Cryptococcus*, healthy adult 11 by *Cyberlindnera* (ASV closely related to *C. jadinii* or *Candida utilis*), healthy baby 3 by *Aureobasidium* and *Sporobolomyces*, whereas healthy adult 12 presented a mixed fungal community.

In contrast, the fecal bacteriome was more diverse (Figure S5A). Although the profiles remained stable during the control period, they drastically shifted during the antibiotic treatment period, as illustrated by every α-diversity indicator largely impacted across all donors, with the number of observed ASV decreasing in all donors (Figure S5B and Figure 7C). Yet, upon cessation of antibiotic dosage, most donors recovered their major bacterial taxa, except for healthy adult 12. This is summarized in the principal component analysis (PCA) plot (Figure S5C). While the two time points of the control period cluster together in a donor-dependent manner, all recovery time points fall together away from the control period, indicative of a loss of inter-individual differences.

### 3.3. Specific Bacterial Signatures Are Associated with Changes in Fungal Concentration

Because fungal overgrowth was associated with bacterial diversity loss during the antibiotic treatment period, an exploratory analysis was performed to determine which of the bacterial ASVs correlated the most to the fungal growth resistance. Healthy baby 3 was not considered for this analysis as its bacterial signature largely deviates from the other three healthy adults. The two samples from the control period (C1 and C2) of healthy adults 10, 11, and 12, characterized by a low fungal concentration, were grouped together, and labelled as “low”. The sample during antibiotic period (CLI or CLA) and the first sample during the recovery period (WO1) of healthy adults 10 and 11, characterized by high fungal concentration, were grouped together, and labelled as “high”. The samples CLI and WO1 from healthy adult 12 were not considered because the fungal concentration during the antibiotic and recovery periods remained low. This could be associated with either a lack of fungi or the presence of bacteria exerting anti-fungal resistance that are resilient to the antibiotic treatment.

**Figure 8:**
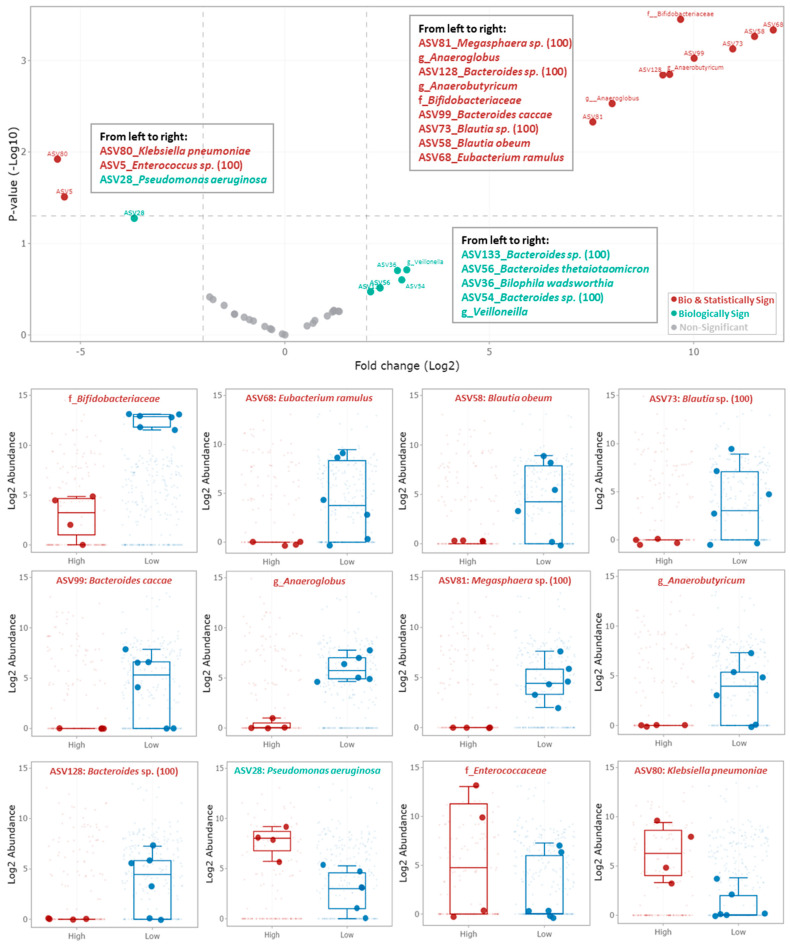
Volcano plot, obtained through differential abundance analysis (treeclimbR), and pairwise analysis using 16S rRNA gene-targeted sequencing data and comparing differences between samples characterized by “low” fungal level (C1 and C2 HA10-11-12) versus “high” fungal level (CLI or CLA and WO1 HA10-11). Samples are considered biologically significantly different if the fold change is higher than 2 log_2_, and statistically significant if the *p*-value of the statistical test is lower than −1.3 log_10_. The name of the species closely related to each ASV is provided. If no species can be assigned, the genus is mentioned with the identity score indicated under brackets. ASV = amplicon sequence variant; Bio = biologically; Sign = significant; f_ = family; g_ = genus.

The abundance of several ASVs was significantly different between the samples with low and high fungal loads (<−2 log_10_ *p*-values) (Figure 8). The family *Bifidobacteriaceae*, the genus *Anaeroglobus*, and a species belonging to the genus *Megasphaera* (ASV81) were always present and highly abundant across all samples characterized by low fungal loads. Their abundance was significantly lower in the samples characterized by high fungal loads. In addition, independent of their prevalence in the samples with low fungal loads, the following ASV (with their closely related species assignment) and genus abundances were significantly lower in the samples characterized by high fungal loads: ASV68 (*Eubacterium ramulus*), ASV58 (*Blautia obeum*), ASV73 (unidentified *Blautia* sp.), ASV99 (*Bacteroides caccae*), ASV128 (unidentified *Bacteroides* sp.), the genera *Anaerobutyricum,* and *Anaeroglobus*. Finally, the family *Enterococcacaea* and the ASV80 (*Klebsiella pneumoniae*) abundances were significantly higher in the samples characterized by high fungal loads, whereas ASV28 (*Pseudomonas aeruginosa*) abundance was biologically (>2 log_2_ fold change) but not significantly higher.

## 4. Discussion

In this study, we embarked on an exploration of the capacity of the L- and M-SHIME^®^ in vitro model to maintain mycobiome abundance and diversity within proximal and distal colonic compartments using fecal microbiota from individuals spanning healthy adults, healthy babies, and those afflicted by active Crohn’s disease. Additionally, we probed the effects of antibiotics on the colonic mycobiome, introducing shifts in bacterial communities and employing quantitative and sequencing methods to dissect mycobiome dynamics within a complex bacterial background.

Our observations unveiled intriguing insights. The fungal concentration within feces from healthy adults exhibited substantial variability, ranging between 3.88 and 7.62 log_10_(18S rRNA gene copy/mL). This aligns with findings from a study involving 221 pregnant women, which reported an average fecal fungal content of 3.85 log_10_(ITS/mL) and values ranging from 0 to 10 log_10_(ITS/mL) [45]. Furthermore, fecal samples from healthy babies and Crohn’s disease patients exhibited higher fungal concentrations than those from healthy adults, although this elevation in concentration did not consistently translate to a higher proportion of observed ITS1 ASVs as compared to 16S ASVs.

Longitudinal tracking of microbiome abundance throughout different SHIME^®^ colonic compartments indicated that fungal concentrations remain stable across the simulated gut. No discernible differences in fungal content emerged between proximal and distal colon reactors, and the presence or absence of a mucosal compartment did not yield divergent fungal profiles. Nonetheless, a direct comparison between the M-SHIME^®^ and L-SHIME^®^ was not feasible in our experimental design and warrants further exploration by running both configurations with microbiota from the same fecal inoculum.

Exploring fungal colonization capability within the simulated gut, we observed consistent absolute fungi concentrations aligned with those in the original fecal inocula. This was particularly apparent in SHIME^®^ reactors with healthy adults inocula, where low initial fungi levels correlated with sustained low levels within the colon reactors. As an exception and interestingly, healthy adult 7 was characterized by a high fecal fungal concentration but displayed a pronounced decline in the fungal load during the SHIME^®^ stabilization phase. This decrease was attributed to the dominance of *Debaryomyces* (ASV closely related to *D. hansenii*) in the fecal inoculum, a fungus known to contaminate foods with low water activity, such as cheese. It is also widely used in the food industry [46]. It can, therefore, be present in the gut of individuals consuming these food products. While *Debaryomyces* could not engraft due to its inability to grow at 37 °C [13], and the absence of consistent inoculation through the SHIME^®^ feed, its decline might have created an ecological and functional niche that allowed *Trichosporon* to thrive. *Trichosporon* spp. are a genus known to colonize the human gut, leading to systemic infections in patients with predisposing factors such as hematological cancer [47,48]. The lack of an immune component in our SHIME^®^ model may have contributed to the outgrowth of this opportunistic pathogen.

While experiments with the microbiota from healthy individuals displayed low fungi levels, SHIME^®^ colon compartments from Crohn’s disease patients showed higher fungal concentrations, quite consistent with their original fecal inoculum. Yet, profound shifts in fungi composition were observed during the stabilization period in the SHIME^®^. For instance, *Candida* (ASV closely related to *Candida albicans*) dominance in Crohn’s disease patient 2 gave way to *Penicillium* in the proximal colon reactor. *Candida albicans* is a commensal of the human GIT that has co-evolved with warm-blooded mammalians [12]. It exhibits a large carbon utilization versatility, which is a pre-requisite to survive in the GIT where the nutrients available are constantly changing depending on the diet [49]. However, we used a standardized feed composition to simulate the nutritional daily intake of a healthy individual which might not align with its versatile carbon utilization requirements. In addition, the model lacks an immune component that would establish a pro-inflammatory tone during flares, particularly relevant in Crohn’s disease. Therefore, we must acknowledge that the conditions might no longer be ideal for *Candida* to thrive. It is also possible that only dead cells or free DNA of *C. albicans* are present in the fecal samples that ultimately got washed out from the reactors during the stabilization period. This would need further investigation to confirm the ability of this fungus to colonize the SHIME^®^.

Importantly, SHIME^®^ reactors from Crohn’s disease patients had similar bacterial concentration to the ones from healthy adults yet contained higher fungal concentrations. This suggests that total bacterial concentrations do not predict total fungal concentrations. Furthermore, the bacterial α-diversity was lower in the fecal inoculum of Crohn’s disease patients than that of healthy adults, indicative of an impaired (or dysbiosed) bacteriome, as previously reported [50]. This difference may result in lower colonization resistance from the bacteriome against fungi, allowing their expansion.

While experiments involving Crohn’s disease patients exhibited high fungal concentrations as opposed to healthy adults, even greater levels were observed in SHIME^®^ colon reactors populated with fecal samples from the three healthy babies. Notably, the fungal concentrations in these babies’ reactors were either similar to or even higher than their original fecal inoculum. Additionally, the fungal composition in healthy baby 3, initially dominated by *Ramularia*, shifted to *Aureobasidium* and *Sporobolomyces* in the proximal colon reactor. Intriguingly, although rarely diagnosed, recent evidence has linked human systemic infection caused by *Aureobasidium* spp. to catheter usage [51,52] underlining its ability to thrive at body temperature. Similarly, human infections have been associated with *Sporobolomyces* spp. [47,53], highlighting their potential pathogenicity. Therefore, the rise in fungal concentration within certain reactors compared to the initial fecal samples of babies could suggest the presence of more favorable environmental conditions in vitro, such as nutrients availability, or the potential impact of the absence of the immune system.

Remarkably, the fecal inoculum from healthy babies had lower bacterial α-diversity and total bacterial concentrations compared to both healthy adults and Crohn’s disease patients. Given that these babies were 5 to 6 months old, their microbiome were still in an immature state, consistent with prior research [54]. Moreover, the lower total bacterial concentrations might create a larger nutrient pool, potentially promoting heightened fungal growth. Consequently, these findings reaffirm the pivotal role of a reduced bacterial α-diversity in diminishing bacterial colonization resistance against fungi, thereby allowing fungal establishment. Furthermore, a secondary role emerges from the low total bacterial concentration, contributing to the escalation of total fungal concentration.

Recognizing the significance of bacteriome-mediated colonization resistance against fungi, we delved into the impact of antibiotic treatments on fungal dynamics. These antibiotics were administered to colon compartments where the microbiome from healthy adults or babies had reached a stabilized state, implying that colonization resistance was at its peak at the outset of the treatment. Two distinct antibiotic treatments were employed, namely clindamycin and a combination of amoxicillin and clavulanic acid. Clindamycin, a lincosamide antibiotic, exhibits activity against a broad spectrum of aerobic Gram-positive cocci and a variety of anaerobic Gram-positive and Gram-negative bacteria [55]. On the other hand, amoxicillin, a semi-synthetic penicillin, is coupled with clavulanic acid, a β-lactamase inhibitor [56]. These antibiotics in tandem lead to a restructuring of the microbial landscape, ultimately fostering the growth of *Enterobacteriaceae* in humans, mice, and dogs [57,58].

The response to antibiotic treatments exhibited variations based on the individual donors. Among the colonic reactors containing microbiota from healthy adults, with the exception of donor 13, the antibiotic application did not exert any discernible influence on the total bacterial concentration. Conversely, the introduction of antibiotics led to a substantial decrease in the overall bacterial concentrations within the reactors that harbored the microbiomes of the three healthy babies. Notably, this outcome did not show a correlation with the specific type of antibiotic dosed. However, it is possible that this effect is linked to the early developmental stage of the baby bacteriome, which displays lower resilience against antibiotics compared to the more mature microbiomes of adults [59].

While most of the SHIME^®^ colonic reactors that contained healthy adult microbiota exhibited only minor shifts in total bacterial concentrations following antibiotic treatment, a large increase in total fungal concentrations was observed in three out of seven donors. Interestingly, this discrepancy in response did not seem to be correlated with the specific antibiotic type employed. Instead, it can be explained by the initially low fungal concentrations (almost at the limit of quantification) before antibiotic introduction (during the control period) in the four healthy adults who showed no notable fungal changes. This implies the possibility that either fungi were absent from the reactors, potentially yielding false positive qPCR results, or that the induced perturbation in the bacteriome was not sufficiently pronounced to allow fungal proliferation.

In contrast, both antibiotic treatments prompted fungal overgrowth in the colonic reactors of three healthy adults and the three healthy babies. It is noteworthy that in the colonic reactors inoculated with two of the three healthy adults, the initial fungal levels were low, nearly at the limit of quantification, prior to the antibiotic administration. This observation carries significance, suggesting that individuals characterized by initially low fungal abundance might still harbor a fungal quorum sufficient to trigger overgrowth in response to antibiotic treatment. This underscores a limitation in the accuracy of molecular-based methods for quantifying fungi, particularly in the context of healthy individuals, wherein the mycobiome constitutes a mere 0.1% of the overall GIT biosphere [5]. Furthermore, higher fungal growth was observed in the proximal colon as compared to the distal colon. This disparity could be attributed to the following: (1) A range of favorable environmental conditions in the proximal region such as lower pH (5.75–5.95 vs. 6.60–6.90 for the proximal vs. distal colon, respectively) and higher carbohydrate availability; (2) Ecological conditions whereby bacterial diversity differs between the two colonic regions (carbohydrate versus proteolytic fermentation) [60].

We previously underscored the significance of a healthy adult bacteriome in furnishing colonization resistance against fungi, contrasting with the resistance arising from compromised microbiomes in Crohn’s disease patients or immature microbiomes in babies. Through our antibiotic experiments, it became evident that the escalation in fungal concentrations following antibiotic administration was not solely linked to diminished bacterial concentrations. This observation reaffirmed the concept that by reshaping the bacteriome profile it becomes plausible to modulate the extent of colonization resistance. Thus, a profound interplay between fungi and bacteria is discernible. Remarkably, while the fungal α-diversity remained mostly stable, the bacterial α-diversity was curtailed post-antibiotic treatment, in tandem with a decline in inter-individual variations within the bacteriome and observed ASV counts. By reducing the α-diversity, antibiotics can foster an environment conducive to diminished colonization resistance of the bacteriome against fungi, paving the way for opportunistic agents, such as fungi, to proliferate [15]. Interestingly, in our study, the four fungal genera that appeared to benefit from the reduced colonization resistance are recognized as potential human pathogens. Apart from *Sporobolomyces* and *Aureobasidium*, both implicated in human ailments, *Cyberlindnera* (ASV closely related to *C. jadinii* or *Candida utilis*) is also frequently linked to GIT infections in humans [61,62,63,64]. Furthermore, *Cryptoccocus* spp., albeit infrequently detected in the human colon, has been documented to cause infections in humans [65,66]. The implication of these findings, where fungal pathogens thrive in the microbiome post-antibiotic treatment, might signal the utility of SHIME^®^ in vitro findings in predicting in vivo outcomes.

Given the direct correlation between the rise in fungal levels and the antibiotic dosage in these donors, it became pertinent to scrutinize the bacteriome before, during, and after treatment. Such analysis could potentially uncover distinctive bacterial signatures that corresponds to shifts in fungal load. Our investigation unveiled a significant reduction in the *Bifidobacteriaceae* family across all donors with elevated fungal concentrations. Our findings align with recent research, both in vitro [67] and in vivo [68,69], suggesting that *Bifidobacterium* spp. possess antagonistic properties against fungi, including *Candida albicans*. Importantly, evidence exists of species- and strain-specific anti-*Candida* activities within the *Bifidobacterium* genus. This phenomenon could elucidate the disparities between babies, where an ASV closely related to *B. catenulatum* dominates, and adults, where an ASV closely related to *B. adolescentis* prevails. Notably, *B. adolescentis* demonstrated the strongest anti-*Candida* activity among all tested *Bifidobacterium* species in vitro [67]. However, additional research is warranted to verify whether *B. adolescentis* can indeed restrain fungal growth in vivo, while excluding other potential confounding variables. Furthermore, we observed a notable reduction in *Bacteroides* and *Megasphaera* in samples marked by elevated fungal concentrations. *Bacteroides* is known to degrade specific components of the fungal cell wall, such as chitin and mannose [20,70]. Additionally, *Megasphaera* was previously found to negatively correlate with fungal proliferation in keratitis patients [71]. While acknowledging the limitations in statistical power, this preliminary analysis provides a foundation for hypothesis formulation and highlights the utility of the SHIME^®^ model not only in comprehending inter-kingdom dynamics but also in identifying bacteria with potential probiotic attributes.

Finally, we delved whether the fluctuations in fungal dynamics correlated with the levels and profiles of common fermentation byproducts, including short-chain fatty acids (SCFAs). These bacterial metabolites are often associated with exhibiting anti-fungal attributes [72,73]. However, within the physiological context of the SHIME^®^, it appeared that acetate, propionate, and butyrate did not exhibit discernible anti-fungal properties. Despite the SCFA concentrations within reactors, inoculated with microbiota obtained from babies or adults, being notably elevated and comparable to those observed in vivo, these same reactors harbored elevated fungal levels. This observation suggests that SCFA might have species-specific effects on fungi. Alternatively, it would imply that more simplified experimental models, such as batch fermentations using RPMI, PBS, or YPD media under aerobic growth conditions and without microbiome background [72,73], might have limited predictive power. Such limitation was recently exemplified by McDonough and colleagues whereby *Candida albicans* strain 529L exhibited a filamentation defect in vitro but not in vivo [74]. Consequently, it underscores the critical necessity of employing robust in vitro methodologies when studying fungi, as their behavior in simpler settings might not accurately reflect their behavior in more complex biological environments. Nonetheless, considering the pivotal role of bacterial α-diversity in influencing fungal engraftment and overgrowth, other bacterial metabolites aside from SCFA could have notable toxicological effects. For instance, secondary bile acids like lithocholic acid and deoxycholic acid have demonstrated inhibition of *C. albicans* growth in vitro [75]. Therefore, further investigations utilizing metabolomics in conjunction with the SHIME^®^ could provide a deeper mechanistic understanding of the interaction between fungi and bacteria.

To the best of our knowledge, this study represents a pioneering effort in amalgamating quantitative and sequencing methodologies to investigate the inter-individual dynamics of both the mycobiome and bacteriome. Our investigation spanned 21 distinct fecal samples and harnessed a semi-continuous fermentation model of the human GIT, while also considering the presence or absence of antibiotic treatments. It is notable that Payne and colleagues had embarked on a similar path back in 2003 [76] utilizing a MacFarlane-based fermentation model [77] for 48 days to explore the interplay between *Candida albicans* and a probiotic strain, which was then called *Lactobacillus plantarum* LPK (now *Lactiplantibacillus plantarum*). This earlier work involved a fresh fecal microbiome, and it included a unique tetracycline dose administered on day 29. They used selective growth medium to enumerate some major bacterial taxa, thereby showcasing the beneficial effect of the probiotic dosage in reducing *C. albicans* growth. In 2004, the same research group continued their exploration [78], this time delving into the dynamics of the bacteriome and mycobiome over a seven-day duration. This experiment involved treatments with tetracycline (antibacterial) and nystatin (antifungal). Intriguingly, both studies evidenced that tetracycline treatment correlated with escalated fungal concentrations, thus corroborating findings from our own study. More recently, Maas and colleagues also engaged in investigating the interplay between bacteria and fungi using the TIM-2 fermentation model [79,80]. Their focus encompassed diet modulation as well as the application of antifungal and antibacterial treatments. They used sequence-based methods to track microbial dynamics over a span of 72 hours subsequent to 16- or 20-hour adaptation periods. However, their approach of pooling fecal samples from various individuals inadvertently omitted the opportunity to examine inter-individual disparities in these inter-kingdom interactions. Furthermore, the absence of cell quantification (whether culture-dependent or qPCR-based) or the assessment of cell viability (through flow cytometry or by treating samples with propidium monoazide before DNA extraction), particularly relevant in such short study period, posed limitations to data interpretation. This shortfall was particularly evident in our study where the wash-out of *Debaryomyces hansenii* and *Candida albicans* from the reactors highlighted the importance of considering these aspects.

## 5. Conclusions

In this study, we have effectively harnessed the capabilities of the SHIME^®^ system to comprehensively investigate the dynamics of both the mycobiome and bacteriome over an extended period of 45 days, encompassing conditions of both eubiosis and dysbiosis, while acknowledging the limitations in statistical power. This investigation involved a total of 21 diverse fecal samples, representing healthy adults, healthy babies, and diagnosed Crohn’s disease patients with flare-up symptoms. Our findings underscore the remarkable inter-individual variability in fungal concentrations. Healthy adults exhibited notably lower fungal concentrations, while higher concentrations were evident in cases of an immature microbiome (babies) or a disrupted one (Crohn’s disease patients). This observation lends further weight to the proposition of the bacteriome’s pivotal role in resisting fungal colonization. The intricate interplay between fungi and bacteria was further probed through the application of two antibiotic treatments. Our study revealed a crucial nuance: relying solely on total bacterial concentrations cannot anticipate the extent of fungal engraftment. Instead, it appears that once predisposing conditions align, a lower bacterial content facilitates heightened fungal growth, plausibly due to the availability of greater nutrients. This predisposition is notably correlated with the composition of bacterial community. We found that the concentrations of *Bifidobacteriaceae*, *Bacteroides*, and *Megasphaera* were inversely linked to fungal concentrations. Strikingly, the fungal genera that thrived amidst bacterial dysbiosis consisted exclusively of opportunistic pathogens, which have been associated with documented instances of infections in humans. On the whole, the findings from the SHIME^®^ underscore the complexity of fungal colonization success, which is inherently influenced by inter-individual factors and often transient in nature. Furthermore, our study highlights that the capacity of bacterial community to resist fungal overgrowth is primarily tied to specific bacteria genera, rather than merely the overall bacterial load. Finally, while our focus has been on fungi–bacteria interactions, delving into mycobiome–virome interplay could unveil additional holistic insights into how fungi interact with other community members present in the gastrointestinal tract.

## Data Availability

The raw sequences and corresponding metadata can be consulted upon request to the authors.

## Supplementary Materials

The following supporting information can be downloaded at: https://www.mdpi.com/article/10.3390/jof9090877/s1, Figure S1: Total bacterial concentration in the fecal inocula and during stabilization (day 3 and day 10) in the SHIME^®^ in eubiosis conditions, measured using qPCR targeting the 16S rRNA gene region and expressed in log_10_ (16S rRNA gene copy/mL); Figure S2: Bacterial quantitative microbiome profiling (A), and α-diversity indexes (B) in fecal samples and resulting proximal colon reactors during stabilization in eubiosis conditions. Discriminant analysis of principal components (C) for β-diversity visualization; Figure S3: Total bacterial concentration in the SHIME^®^ during eubiosis conditions (control period = C1, C2), during antibiotic treatment (=AB), and during the recovery period (wash-out = WO1, WO2) measured using qPCR targeting the 16S rRNA gene region and expressed in log_10_(16S rRNA gene copy/mL); Figure S4: Acetate, propionate, and butyrate concentrations in the distal colon reactors during eubiosis conditions (control period = C1, C2), during antibiotic treatment (=AB), and during the recovery period (wash-out = WO1, WO2); Figure S5: Bacterial quantitative microbiome profiling (A) and α-diversity indexes (B) in the proximal colon reactors during eubiosis conditions (control period = C1, C2), during antibiotic treatment [clindamycin (= CLI) or a mix of amoxicillin:clavulanic acid (=CLA)] and during the recovery period (wash-out = WO1, WO2). Principal component analysis plot (C) for visual distribution of the samples.
