# Supplementary material for: The Intricate Connection between Bacterial α-Diversity and Fungal Engraftment in the Human Gut of Healthy and Impaired Individuals as Studied Using the In Vitro SHIME® Model
Source: J Fungi (Basel). 2023 Aug 26;9(9):877. doi: 10.3390/jof9090877 (PMC10532570; doi:10.3390/jof9090877)
Supplement: Supplementary file 1 [file jof-09-00877-s001.zip › jof-2539219-supplementary.pdf]

Supplementary Materials

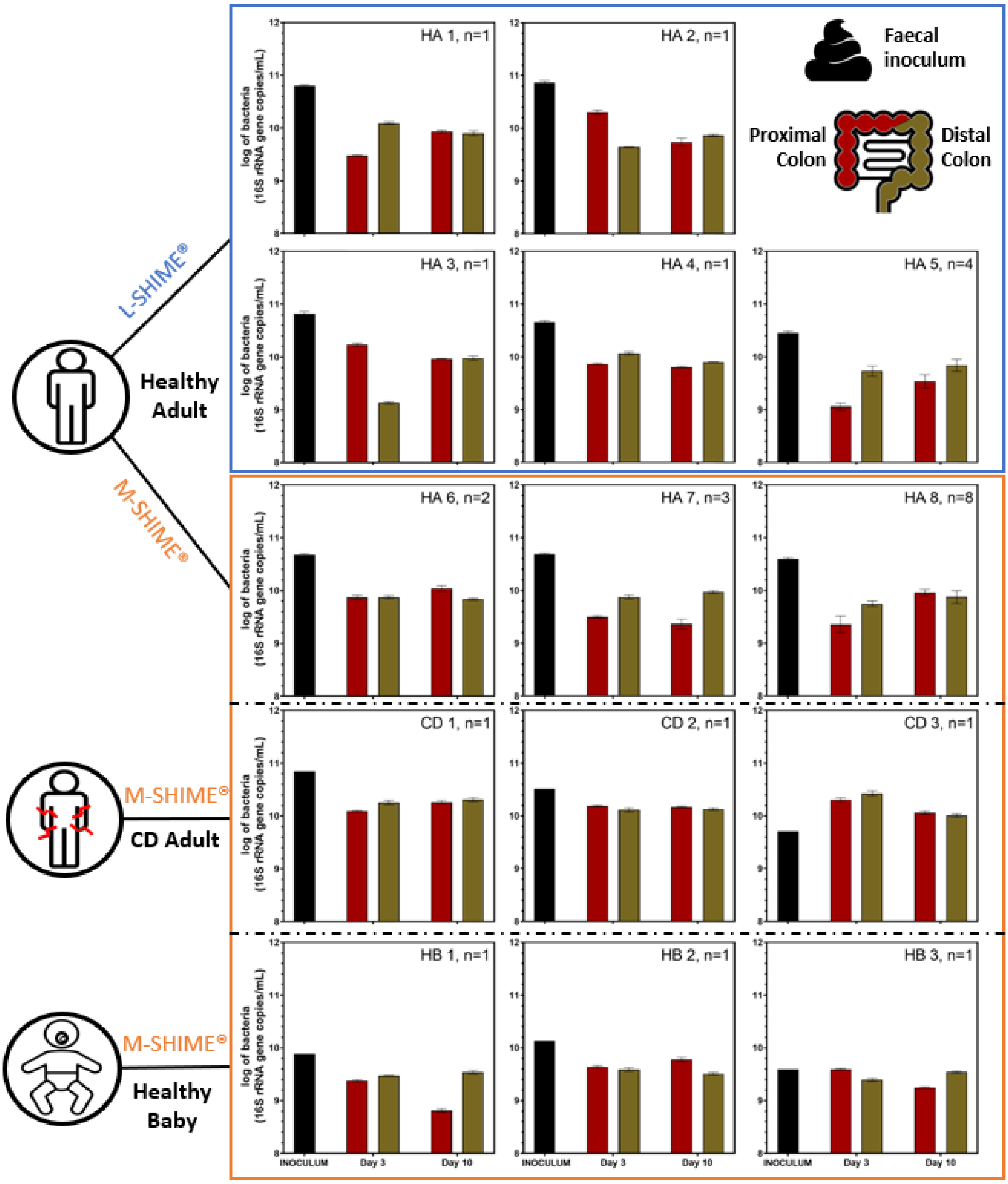

Figure S1. Total bacterial concentration in the faecal inocula and during stabilization (day 3 and day 10) in the SHIME® in eubiosis conditions, measured using qPCR targeting the 16S rRNA gene region and expressed in $\log_{10}$(16S rRNA gene copy/mL). The number of biological replicates for each donor is indicated by the n values, and the analysis was performed with technical triplicates of measurement. The limit of quantification is indicated with a black dashed line. HA = healthy adults; CD = Crohn's disease patient with flare-up symptoms; HB = healthy babies.

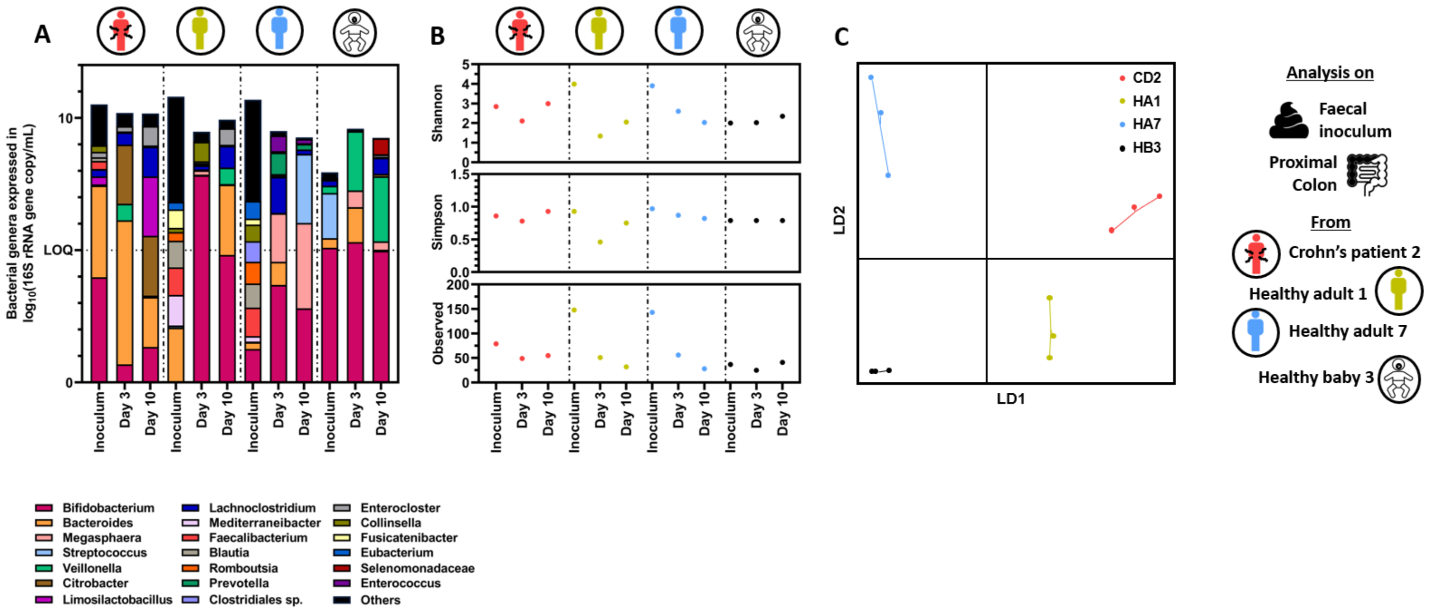

Figure S2. Bacterial quantitative microbiome profiling (A), and α-diversity indexes (B) in faecal samples and resulting proximal colon reactors during stabilization in eubiosis conditions. Discriminant analysis of principal components (C) for β-diversity visualization. For the bacterial quantitative microbiome profiling, each bar represents the total bacterial concentration in the sample, whereas the colors represent the proportion of each bacterial genera. ASV = amplicon sequence variant.

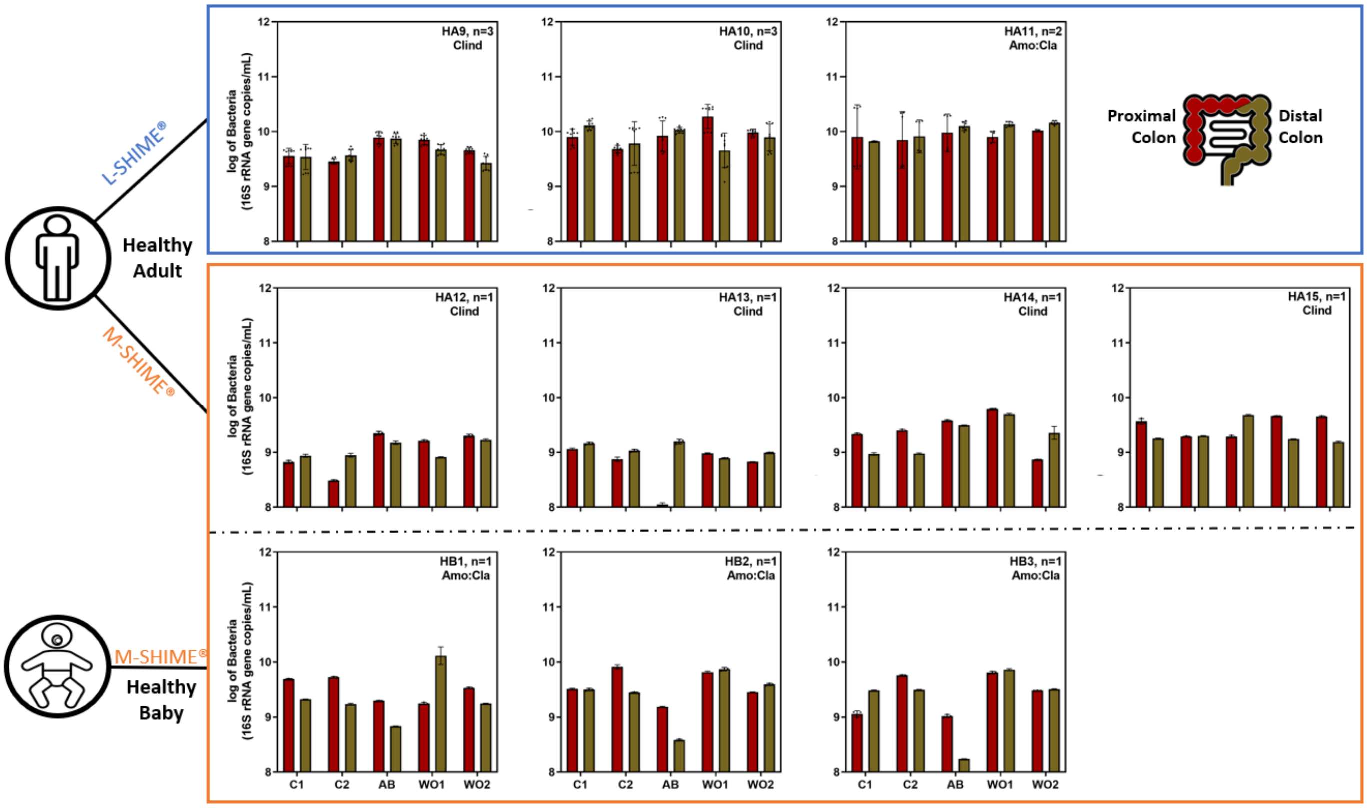

Figure S3. Total bacterial concentration in the SHIME® during eubiosis conditions (control period = C1, C2), during antibiotic treatment (= AB) and during the recovery period (wash-out = WO1, WO2) measured using qPCR targeting the 16S rRNA gene region and expressed in log10(16S rRNA gene copy/mL). The number of biological replicates for each donor is indicated by the n values, and the analysis was performed with technical triplicates of measurement. The limit of quantification is indicated with a black dashed line. HA = healthy adults; HB = healthy babies; Clind = clindamycin; Amo:Cla = amoxicillin:clavulanic acid mix.

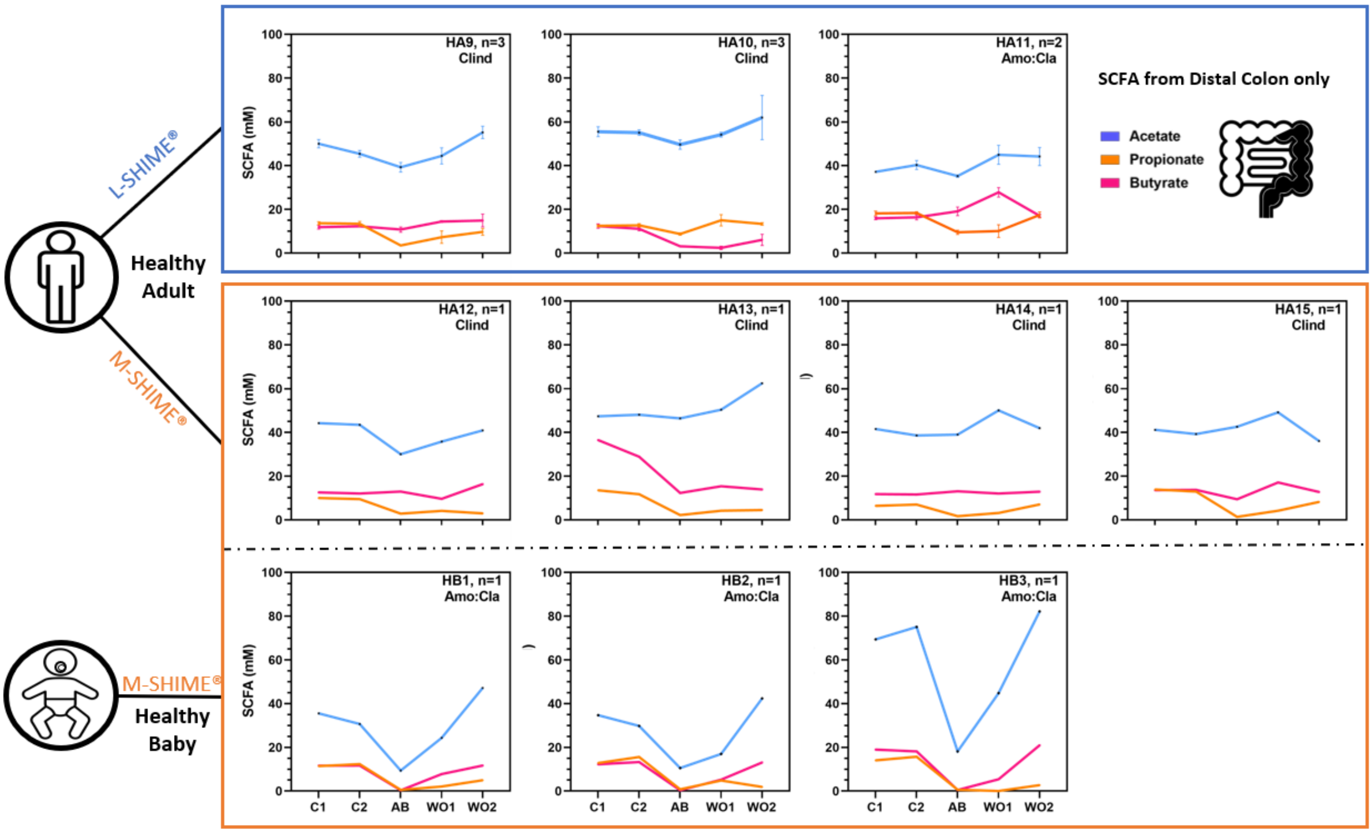

Figure S4. Acetate, propionate, and butyrate concentrations in the distal colon reactors during eubiosis conditions (control period = C1, C2), during antibiotic treatment (= AB) and during the recovery period (wash-out = WO1, WO2). The number of biological replicates for each donor is indicated by the n values, and the analysis was performed with single measurement. HA = healthy adults; HB = healthy babies; Clind = clindamycin; Amo:Cla = amoxicillin:clavulanic acid mix; SCFA = short-chain fatty acid.

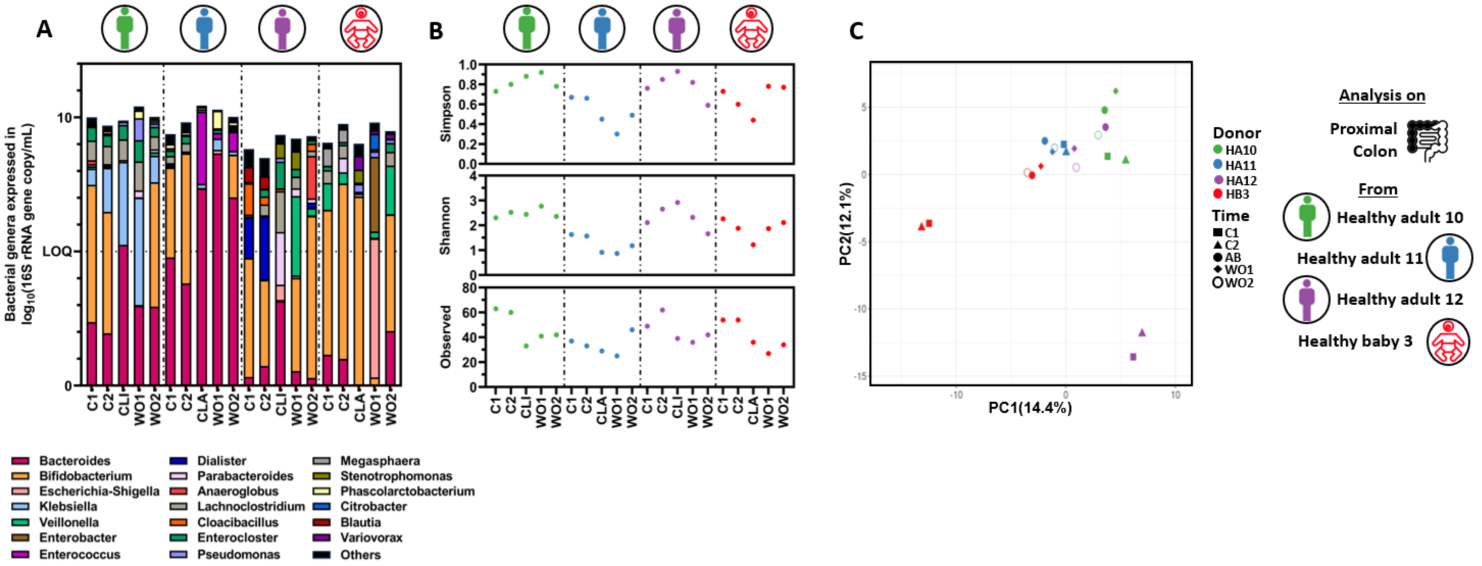

Figure S5. Bacterial quantitative microbiome profiling (A), and α-diversity indexes (B) in the proximal colon reactors during eubiosis conditions (control period = C1, C2), during antibiotic treatment [clindamycin (= CLI) or a mix of amoxicillin:clavulanic acid (= CLA)] and during the recovery period (wash-out = WO1, WO2). Principal component analysis plot (C) for visual distribution of the samples. For the bacterial quantitative microbiome profiling, each bar represents the total bacterial concentration in the sample, whereas the colors represent the proportion of each bacterial genera. ASV = amplicon sequence variant.
